# Binding Specificity of the G1/S Transcriptional Regulators in Budding Yeast

**DOI:** 10.1371/journal.pone.0061059

**Published:** 2013-04-04

**Authors:** Michael R. Harris, Dave Lee, Sarah Farmer, Noel F. Lowndes, Robertus A. M. de Bruin

**Affiliations:** 1 MRC Laboratory for Molecular Cell Biology, University College London, London, United Kingdom; 2 The UCL Cancer Institute, University College London, London, United Kingdom; 3 Centre for Chromosome Biology, Genome Stability Laboratory, School of Natural Science, National University of Ireland Galway, Galway, Ireland; University of Cambridge, United Kingdom

## Abstract

**Background:**

G1/S transcriptional regulation in the budding yeast *Saccharomyces cerevisiae* depends on three main transcriptional components, Swi4, Swi6 and Mbp1. These proteins constitute two transcription factor complexes that regulate over 300 G1/S transcripts, namely SBF (Swi4-Swi6) and MBF (Mbp1-Swi6). SBF and MBF are involved in regulating largely non-overlapping sets of G1/S genes via clearly distinct mechanisms.

**Methodology/Principal Findings:**

Here we establish and confirm protein-protein and protein-DNA interactions using specific polyclonal antisera to whole Swi6 and to the C-terminal domains of related proteins Swi4 and Mbp1. Our data confirm the protein-protein binding specificity of Swi4 and Mbp1 to Swi6 but not to each other, and support the binding specificity of the transcriptional inhibitor Whi5 to SBF and of the corepressor Nrm1 to MBF. We also show the DNA binding preference of Swi4 to the *CLN2* promoter and Mbp1 to the *RNR1* promoter, while Swi6 binds both promoters. Finally, we establish the binding dynamics of Swi4 and Whi5 to the *CLN2* promoter during the cell cycle.

**Conclusions/Significance:**

These data confirm the binding specificity of the G1/S transcriptional regulators. Whereas previous observations were made using tagged Swi4, Swi6 and Mbp1, here we use specific polyclonal antisera to reestablish the protein-protein and protein-DNA interactions of these G1/S transcriptional components. Our data also reveal the dynamic changes in promoter binding of Swi4 during the cell cycle, which suggests a possible positive feedback loop involving Swi4.

## Introduction

G1/S transcriptional regulation has been extensively studied in the budding yeast *Saccharomyces cerevisiae* and the role of the transcription factors and their coregulators are well established [1], [2], [3], [4], [5], [6], [7], [8], [9], [10], [11], [12]. The main G1/S transcription factor components, Swi4, Swi6 and Mbp1, form two heterodimeric transcription factor complexes. A common Swi6 subunit plus one of the DNA binding proteins Swi4 or Mbp1 constitute SBF and MBF respectively. The DNA binding component Swi4 targets SBF to G1/S target promoters via specific association with a recognition sequence named SCB (CGCGAAA), and Mbp1 targets MBF to MCB (CGCGT) sites. Over 300 G1/S transcripts depend on SBF and/or MBF for their periodicity [7], [13], [14], [15]. The genes regulated by both can be further grouped into targets bound by both at the same time and switch genes, where an SBF-to-MBF switch takes place during the G1-to-S transition [16], [17].

Whereas the patterns of expression of SBF and MBF-dependent targets are similar, the mechanisms of regulation are very different. SBF is a transcriptional activator, required to activate G1/S transcription during G1, while MBF is a transcriptional repressor, repressing transcription outside of G1 [1], [7]. This difference in function is most obvious when either Swi4 or Mbp1 is deleted, inactivating SBF or MBF respectively. Inactivation of SBF results in constitutive low expression of its targets, while *mbp1Δ* cells display constitutively high levels of MBF-dependent transcription. Furthermore, the molecular mechanisms involved in the activation and inactivation of SBF and MBF-dependent transcription are distinctly different. SBF-dependent transcription is kept inactive in G1 by the binding of the transcriptional inhibitor Whi5 [4], [7]. Accumulation of Cln3/CDK during G1 results in phosphorylation of Whi5, releasing it from SBF at promoters and shuttling it out of the nucleus. This initiates transcription and results in the accumulation of additional G1 cyclins, Cln1 and Cln2, which, in a positive feedback loop, leads to complete phosphorylation of Whi5 [18]. Subsequent accumulation of Clb/CDK activity during the G1-to-S transition results in the phosphorylation of SBF, which releases it from promoters, turning off SBF-dependent transcription [1], [8], [19]. Conversely, MBF-dependent repression during the G1-to-S transition depends on the MBF co-repressor Nrm1 [7]. Nrm1, a G1/S target itself, accumulates once cells transit into S phase, binds to MBF and represses transcription, forming a negative-feedback loop.

Here, we raise antibodies against the C-terminal domains of related proteins Swi4 and Mbp1 and against full length Swi6. Using these antibodies, we confirm the Swi4-Swi6 and Mbp1-Swi6 interactions and the specific binding of Nrm1 and Whi5 to MBF and SBF, respectively, in a single culture. In addition, we confirm the binding preference of Swi4 for the promoter of SBF target *CLN2* and of Mbp1 for the promoter of MBF target *RNR1* and establish the binding dynamics of Swi4 and Whi5 to the *CLN2* promoter during the cell cycle.

## Materials and Methods

### Strains used in this study

Strains used in this work were generated by standard genetic methods and derived from 15Daub (MATa *ade1 leu2-3,112 his2 trp1-1 ura3Δns bar1Δ*). All yeast strains used in this study are described in Table 1.

**Table 1:** Yeast strains used in this study.

Strain	Genotype	Source
RBY1	MATa, ade1, leu2-3, 112 his2, trp1-1, ura3Δns, bar1Δ	[9]
RBY206	SWI4-6xmyc::KANr	[7]
RBY91	SWI6-6xmyc::URA3	[9]
RBY312	MBP1-13xmyc::TRP1	[16]
RBY205	MBP1-13xmyc::URA3	[7]
RBY207	NRM1-13xmyc::URA3	[7]
RBY46	WHI5-13xmyc::KANr	[7]
RBY467	swi6::TRP1, WHI5-TAP::KANr SWI4-6xMyc::URA3	[7]
RBY124	mbp1::LEU2	[7]
RBY125	swi4::KANr	[7]

### Antibody generation

DNA fragments encoding the C-terminal portions of Swi4 (residues 683–1092) and Mbp1 (residues 632–833) or full length Swi6 were cloned in-frame into the HIS-tag vector pET21c and transformed into the BL21 *E. coli* strain. Peptides were purified by passing lysate over a nickel-agarose affinity column and used to immunize rabbits. The Swi6, Swi4 and Mbp1 antibodies used in this study are freely available upon request.

### Cell synchronization

Mating pheromone arrest synchrony experiments were carried out as described [20].

### Western blotting and co-immunoprecipitation

Whole cell lysates for western blotting were prepared by post-alkaline extraction, entailing a 5-minute room temperature incubation in 100mM NaOH prior to resuspension and boiling for 3 minutes in SDS sample buffer. For each immunoprecipitation, exponentially growing cells were mechanically disrupted in lysis buffer (50 mM Tris-HCl pH 7.5, 1% Triton X-100, 250 mM NaCl) containing protease inhibitors (Complete Mini, Roche) and phosphatase inhibitors (Phosphatase Inhibitor Cocktail 1, Sigma-Aldrich) by 20 minutes vortexing with glass beads (BioSpec) at 4°C. Subsequently, components were immunoprecipitated with anti-Mbp1, anti-Swi4 or anti-Swi6 polyclonal sera by incubating lysates for 2 hours at 4°C with 50 µl 50% protein A Sepharose beads. SDS sample buffer was added to protein purified on beads. Mbp1, Swi4 and Swi6 were detected using the previously described antisera and myc-tagged proteins detected using anti-myc antibody (9E10, Santa Cruz Biotechnology). To minimize interference from immunoprecipitated rabbit immunoglobulin heavy and light chains, pull-downs with anti-rabbit antibodies were carried out using the TrueBlot® anti-rabbit Ig IP beads and secondary antibody. TrueBlot® enables detection of protein bands which would otherwise be obscured by the presence of reduced and denatured heavy and light chain immunoglobulins.

### ChIP analysis

Chromatin immunoprecipitations were performed as described in [21] or as described by [22] and [23]. 3 µl of each antisera was used per ChIP.

### Reverse transcriptase (RT) and quantitative (q)PCR

Total RNA was isolated using the RNeasy kit (Qiagen). The QuantiTect SYBR Green PCR kit (Qiagen) was used for quantitative PCR on ChIP samples and the QuantiTect SYBR Green RT–PCR kit (Qiagen) was used for RT–PCR experiments. Reactions were run on the Chromo-4 qPCR I system (MJ Research) using standard PCR and RT-PCR conditions. Data were analyzed using MJ Opticon Monitor Analysis Software 3.0.

## Results

### Specific antibodies to Swi4, Swi6 and Mbp1

**Figure 1:**
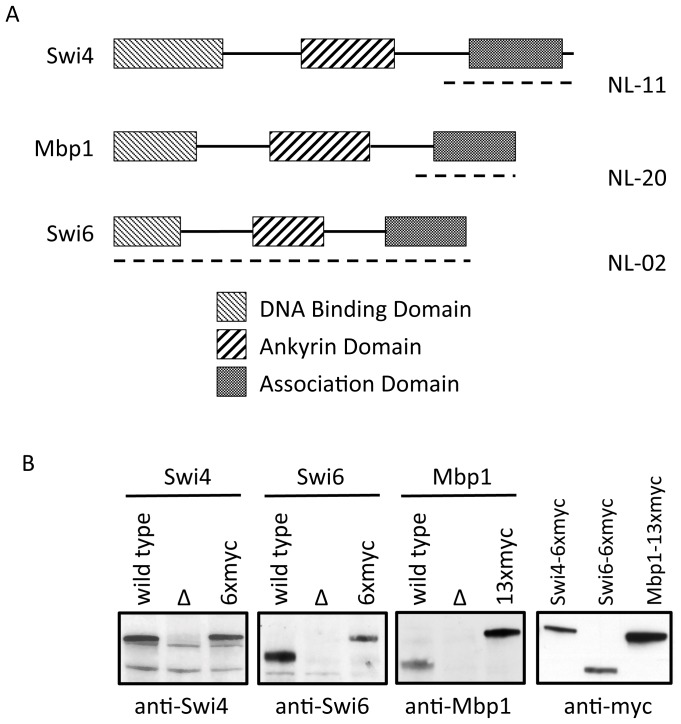
Polyclonal antisera generated against G1/S transcription factor components. (**A**) Regions of functional domains in the three G1/S transcription factor components are represented by the boxed regions. Peptides (dashed lines) from the C-terminal regions of Swi4 (NL11) and Mbp1 (NL20) and full length Swi6 (NL02) were used to immunize rabbits and the resultant polyclonal antisera tested (**B**). Whole cell lysates of asynchronous wild type (wild type), Swi4, Swi6 or Mbp1 deleted (Δ) and Swi4, Swi6, or Mbp1 myc-tagged (6xmyc or 13xmyc) cultures were resolved. Antisera to detect Swi4, Swi6, Mbp1 or myc tagged versions of these components were used as indicated.

In order to study the protein-protein and protein-DNA interactions of the three major G1/S transcription factor components, Mbp1, Swi4 and Swi6, within a single culture, we raised polyclonal sera from rabbits against the C-terminal regions of Mbp1 and Swi4 and against whole Swi6 protein (Fig.1A). To verify the specificity of these polyclonal antibodies, we probed, via western blot, the whole cell lysate of asynchronous cultures of wild type, mutant and myc-tagged strains of each transcription factor component (Fig. 1B). Western analysis indicates that the polyclonal antisera NL02, NL11 and NL20 recognize Swi6, Swi4 and Mbp1, respectively. NL02 identifies Swi6 with an apparent molecular weight of 100 kDa, NL20 identifies Mbp1 as a diffuse band at approximately 120 kDa and NL11 identifies Swi4 with an apparent molecular weight of 150 kDa (Fig. 1B). All three antisera recognize both the wild type and myc-tagged forms. Whereas the Swi6 and Mbp1 antibodies seem highly specific, the Swi4 antibody shows some non-specific binding.

### MBF, but not Swi4, pulls down Nrm1

**Figure 2:**
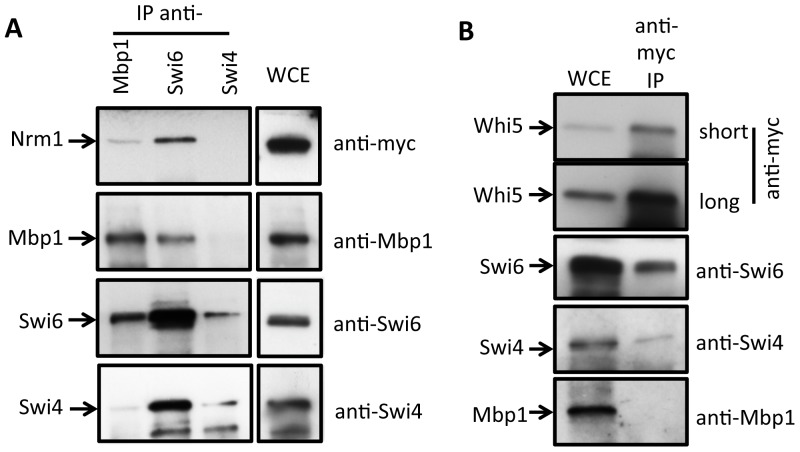
Nrm1 is a component of MBF and Whi5 of SBF. (**A**) Lysate of an asynchronous culture of Nrm1-myc cells was immunoprecipitated using polyclonal antisera against Swi4, Swi6 and Mbp1 (IP anti-Mbp1, Swi6 or Swi4) and analyzed for Nrm1, Mbp1, Swi6, or Swi4 by immunoblotting with anti-myc, anti-Mbp1, anti-Swi6 and anti-Swi4, respectively, as indicated. Whole cell extract (WCE) was immunoblotted with the same antibodies and provided as a control. (**B**) Lysate of an asynchronous culture of Whi5-myc cells was immunoprecipitated using anti-myc antibody (anti-myc IP) and analyzed for Whi5, Swi6, Swi4, and Mbp1 by immunoblotting with anti-myc, anti-Swi6, anti-Swi4, and anti-Mbp1, respectively, as indicated. Long and short exposure of the anti-myc blot is provided. Whole cell extract (WCE) was immunoblotted with the same antibodies and provided as a control.

In order to study the protein-protein interactions of Swi4, Mbp1 and Swi6, we performed immunoprecipitation analyses with each of the polyclonal sera to identify interacting proteins. In addition, we examined their interactions with the MBF co-repressor Nrm1 by performing these analyses on an asynchronous myc-tagged Nrm1 culture (Fig. 2A). Our data show that Nrm1 is co-immunoprecipitated when Swi6 or Mbp1 is pulled down but is not co-immunoprecipitated with Swi4. In summary, Swi6 pulls down Nrm1, Mbp1 and Swi4, whereas Mbp1 pulls down Swi6 and Nrm1, and Swi4 mainly pulls down Swi6. Overall, these results support the binding specificity of Swi4 with Swi6 and of Mbp1 with Swi6 and Nrm1. Although these interactions have been characterized before in great detail, our data support previous observations but here using specific antibodies to the Mbp1, Swi4 and Swi6 components of MBF and SBF in a single lysate.

### Whi5 pulls down SBF not Mbp1

The two concurrently-published papers that initially characterized the role of Whi5 in G1/S transcriptional activation disagreed about whether Whi5 binds MBF in addition to binding SBF [4], [7]. To investigate the binding specificity of the transcriptional inhibitor Whi5 to SBF and/or MBF, we immunoprecipitated myc-tagged Whi5 from an asynchronous culture. Probing this pull-down with anti-Mbp1, anti-Swi4 and anti-Swi6 antibodies shows that only Swi4 and Swi6, but not Mbp1, immunoprecipitate with Whi5-myc (Fig. 2B). These data support a role for Whi5 as an SBF-specific transcriptional inhibitor.

### MBF preferentially binds the RNR1 promoter and SBF the CLN2 promoter

**Figure 3:**
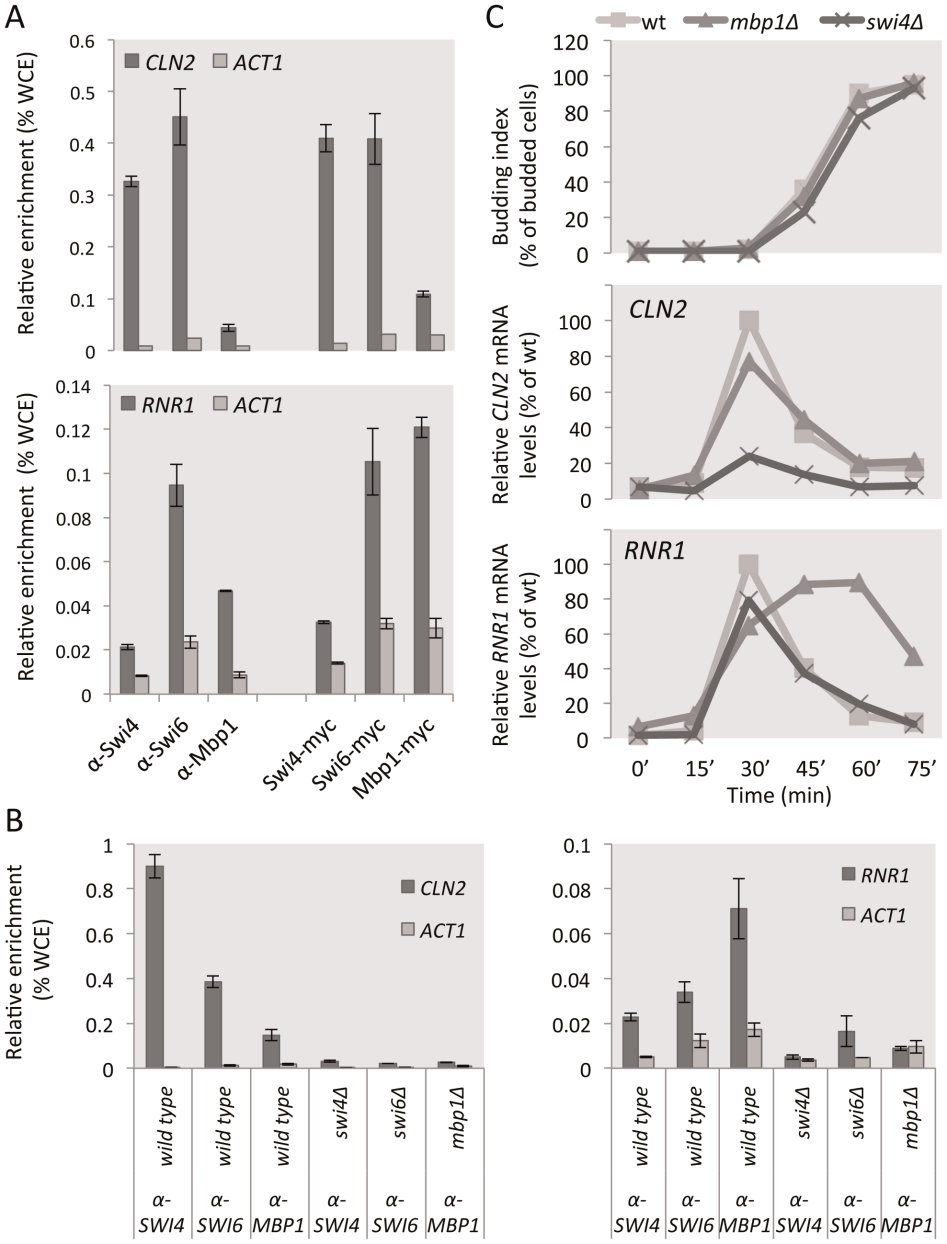
Mbp1 and Swi4 bind and regulate the *RNR1* and *CLN2* promoters respectively. (**A and B**) ChIP analysis for Swi6, Swi4 or Mbp1 at the promoters of *CLN2* (SBF target) and *RNR1* (MBF target). Analysis was performed in asynchronous cells and enrichment levels were assessed by qPCR and normalized to WCE signals (percentage of WCE). *ACT1* signal correspond to non-specific background. Error bars represent standard error calculated from experimental triplicates. (**A**) ChIP analysis was carried out on wild type and myc-tagged Swi4, Swi6 and Mbp1 cell lysates using anti-Swi4, anti-Swi6, anti-Mbp1 or anti-myc antibodies, as indicated. (**B**) ChIP analysis was carried out on wild type and *swi4Δ*, *swi6Δ* and *mbp1Δ* cell lysates using anti-Swi4, anti-Swi6, or anti-Mbp1 as indicated. (**C**) Cultures of indicated strains were synchronized by alpha factor arrest and release. Budding index (% budded cells, upper panel) is provided as an indicator of cell cycle progression. Relative mRNA levels of *CLN2* (SBF target, middle panel) and *RNR1* (MBF target, lower panel) were analyzed by qPCR during the cell cycle in *wt* (dark grey), *mbp1Δ* (medium grey) and *swi4Δ* (light grey) lines. Expression levels are plotted as percentage of highest value detected in wild type experiment (100%).

We next investigated whether the specific polyclonal sera could be used to study the protein-DNA interactions of Mbp1, Swi4 and Swi6. By performing chromatin immunoprecipitations (ChIP), we tested whether the specific polyclonal antisera pull down cross-linked promoter regions of the well-established SBF-dependent *CLN2* promoter and/or the promoter of MBF target gene *RNR1*. We carried out ChIP analysis of wild type cells with the Mbp1, Swi4 and Swi6 specific antibodies, and compared it to anti-myc ChIP analysis of myc-tagged Mbp1, Swi4 and Swi6 strains. Anti-Swi4 sera preferentially enriches pull-downs for the *CLN2* promoter (SBF target) over the *RNR1* promoter (MBF target), whilst anti-Mbp1 sera pull-downs are specifically enriched for *RNR1* and significantly less for *CLN2* promoter regions (Fig. 3B). Anti-Swi6 sera pull-downs contain both promoter regions. These results are similar to those obtained in ChIP analysis using anti-myc antibodies pulling down myc-tagged Mbp1, Swi4 and Swi6, indicating that the antisera are suitable for use in ChIP and can therefore be designated as ‘ChIP grade’. Interestingly, our ChIP data, using anti-myc or the specific antibodies, show binding of Mbp1 to the *CLN2* promoter and of Swi4 to the *RNR1* promoter above *ACT1* background levels. To establish if these signals are Swi4 or Mbp1-dependent, and therefore represent true binding, we carried out ChIP analysis on wild type, *swi4Δ*, *swi6Δ* and *mbp1Δ* cells, using the specific antibodies. These data indicate that there is, in fact, a low level of Swi4 binding to the *RNR1* promoter and of Mbp1 binding to the *CLN2* promoter. To confirm the previously reported dependency of *CLN2* expression on Swi4 and of *RNR1* on Mbp1, we carried out a cell cycle synchrony experiment, using alpha factor block and release, followed by expression analysis of these two G1/S genes in wild type, *swi4Δ* and *mbp1Δ* cells (Fig. 3C). These data show that, while there may be low levels of cross binding, the transcriptional induction of *CLN2* during G1 depends on Swi4 but not Mbp1, whereas the transcriptional repression of *RNR1* outside of G1 depends on Mbp1 but not Swi4.

### Swi4 binding to SBF target promoters is enhanced during G1

**Figure 4:**
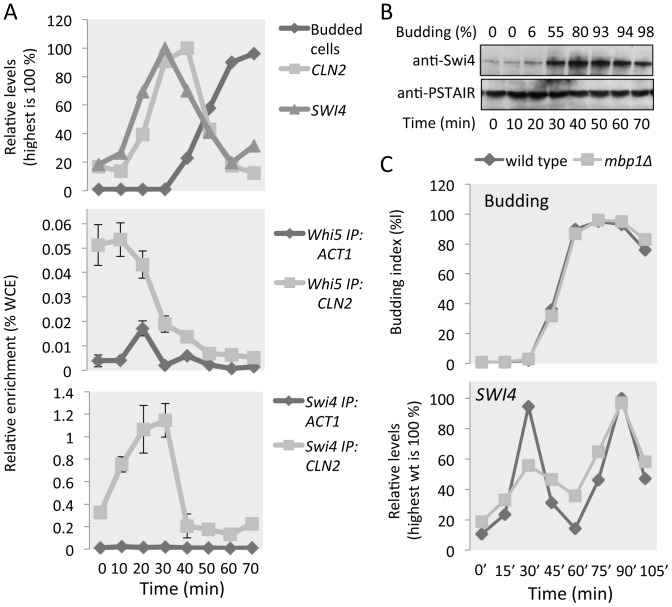
Dynamic changes of SBF target gene regulation during the cell cycle. Cells were synchronized by alpha factor arrest and release. Relative mRNA levels of *CLN2* (SBF target) and *SWI4* (G1/S target) gene expression in synchronized Whi5-myc cells were analyzed by qPCR. Expression levels are plotted as percentage of highest value detected (100%). Budding index (% budded cells, dark grey line, upper panel) is provided as an indicator of cell cycle progression. ChIP analysis for Whi5-myc (light grey line, middle panel) and Swi4 (light grey line, lower panel) binding to *CLN2* during the cell cycle was achieved via anti-myc and anti-Swi4 pull downs. Enrichment levels of pulled down DNA was assessed by qPCR and signals were normalized to WCE signals (percentage of WCE). *ACT1* signal (dark grey line: Whi5 IP, middle panel; and Swi4 IP, lower panel) represents non-specific background. Error bars represent standard error calculated from experimental triplicates and representative data for multiple independent experiments is shown. (**B**) Whole cell lysates of synchronized Whi5-myc cells were resolved. Anti-Swi4 was used to detect Swi4 and anti-PSTAIR to detect Cdc28, shown as a loading control. (**C**) Cultures of indicated strains were synchronized by alpha factor arrest and release. Budding index (% budded cells, upper panel) is provided as an indicator of cell cycle progression. Relative mRNA levels of *SWI4* were analyzed by qPCR during the cell cycle in *wt* (medium grey) and *mbp1Δ* (light grey). Expression levels are plotted as percentage of highest value detected in wild type experiment (100%).

Having established that our antisera are suitable for ChIP, we sought to examine Swi4 binding during the cell cycle to the SBF-dependent *CLN2* promoter and compare this to the binding of SBF component Whi5. Previous studies have shown that Whi5, an inhibitor of SBF-dependent transcription in early G1, leaves promoters just prior to G1/S transcriptional activation [4], [9]. As a target of both the M/G1 cell cycle transcription factor Mcm1 and MBF, *SWI4* expression peaks in early G1 and this regulation is thought to be important for timely activation of G1/S transcription [24], [25]. The possibility that the transcriptional induction of *SWI4* has an effect on Swi4 binding to G1/S promoters during G1 has not been previously assessed by ChIP qPCR. We arrested Whi5-myc cells in G1 with alpha factor, released the arrest and monitored cell cycle-dependent transcription and binding of Whi5 and Swi4 to the *CLN2* promoter at 10-minute intervals (Fig. 4A). Our data show that Swi4 and Whi5 are both bound to the *CLN2* promoter during early G1. Whi5 dissociates from the *CLN2* promoter at 30 minutes, which coincides with transcriptional activation. As expected, Swi4 remains bound to the promoter until transcriptional inactivation and cells start to bud (60 minutes). Interestingly, we detect enhanced binding of Swi4 to promoters after loss of Whi5. This might reflect better antibody recognition of Swi4, or stronger association of Swi4 with the DNA, as a result of loss of the interaction between Whi5 and SBF. Alternatively, since this recruitment precedes activation of G1/S transcription, it might be a direct consequence of the observed Swi4 protein accumulation (Fig. 4B) as a result of transcriptional activation of *SWI4* (Fig. 4A).

Interestingly, *SWI4* expression remains periodic when the Mcm1 binding site, ECB (**E**arly **C**ycle **B**ox), in the *SWI4* promoter is mutated [24], [25]. The mutation only results in a slight delay in peak transcription causing *SWI4* cell cycle expression to coincide with the G1/S wave of transcription. This periodicity is thought to be MBF-dependent based on the presence of an MBF binding site, MCB, in the Swi4 promoter. To test this, we investigated *SWI4* expression in wild type and *mbp1Δ* cells in an alpha factor arrest and release experiment (Fig. 4C). These data show that *SWI4* peak expression largely depends on Mbp1 in the first cell cycle after release from alpha factor block. Expression levels are unaffected in the second cell cycle with peak expression corresponding with the M/G1 transition, just before unbudded cells return into G1. These data show that, after release from alpha factor block, *SWI4* peak expression largely depends on Mbp1, indicating that MBF could be involved in extending the accumulation of Swi4. Together our data suggest a possible positive feedback loop where activation of G1/S transcription results in extended accumulation of Swi4, further recruitment of active SBF to G1/S promoters, thus promoting G1/S transcription.

## Discussion

Here we use specific antibodies to the three main G1/S transcription factor components in budding yeast, Swi4, Swi6 and Mbp1, to confirm and extend previous observations made with tagged versions of these proteins. Our data show that unaltered versions of Swi4, Mbp1 and Swi6 display the same protein-protein and protein-DNA binding specificities as their tagged equivalents. Using Swi4, Swi6 and Mbp1-specific antibodies, we examine the interactions with each other and with the co-regulators Whi5 and Nrm1 in single cultures. We show that some Swi4 can be found at the MBF-dependent *RNR1* promoter and some Mbp1 at the SBF target *CLN2* promoter. Finally, we determine the binding dynamics of Swi4 to SBF target promoters and correlate this with transcription and Whi5 binding in a single culture. These data suggest that SBF might be further recruited to promoters after Whi5 dissociation, coinciding with transcriptional activation of G1/S genes and accumulation of Swi4.

The antisera described here will undoubtedly be useful for future investigation into the cell cycle dynamics of Swi4, Mbp1 and Swi6 protein levels, protein-protein binding, and protein-DNA binding. For example these antibodies have been used in a study to establish the function of a conserved region found in Whi5 and Nrm1, the so called *G*1/S *T*ranscription factor *B*inding (GTB) motif, showing that it is necessary and sufficient for binding SBF and MBF, respectively [26].

**Figure 5:**
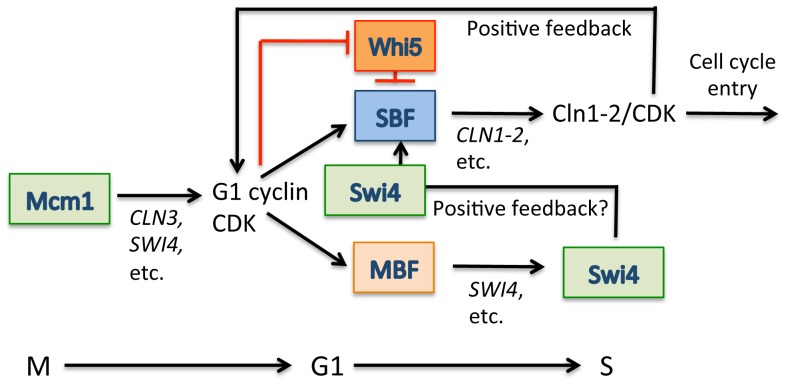
Model of SBF transcriptional regulation. During G1, SBF (Swi4-Swi6) is bound to target promoters in complex with the transcriptional inhibitor Whi5, which represses transcription. Activation of Mcm1-dependent transcription results in the initial accumulation of Cln3 and Swi4. Cln3/CDK-dependent phosphorylation removes Whi5 from SBF at promoters, activating transcription. Initial transcriptional activation results in SBF-dependent accumulation of Cln1-2 and MBF-dependent further accumulation of Swi4. Cln1-2 in complex with CDK is involved in a positive feedback loop to further phosphorylate Whi5, which leads to robust activation of G1/S transcription. Accumulation of Swi4 during G1 coincides with enhanced detection of Swi4 at the *CLN2* promoter, possibly representing an additional positive feedback loop to ensure timely activation of G1/S transcription.

The binding dynamics of myc-tagged Swi6 and Mbp1 during the cell cycle have been previously determined using ChIP analysis followed by qPCR [7]. Mbp1 is constitutively bound to its target promoters throughout the cell cycle, with a slight dip in binding outside of G1. Swi6 is also found at MBF-dependent promoters throughout the entire cell cycle with a more significant loss of binding outside of G1. In contrast, Swi6 is only detected at SBF-dependent promoters during G1 with a complete loss of binding outside of G1. Here we investigated Swi4 binding during the cell cycle by ChIP qPCR and show enhanced binding to the *CLN2* promoter once G1/S transcription is activated, which coincides with the release of Whi5 from SBF and accumulation of Swi4 protein. Mcm1-dependent transcriptional activation of *SWI4* and, amongst others, *CLN3* in early G1 is required to promote timely activation of G1/S transcription [24], [25]. Despite periodic expression of *SWI4* largely depending on MBF, early activation involves Mcm1. We speculate that the enhanced binding, as a result of Swi4 accumulation, could represent further recruitment of active SBF to G1/S target promoters (Figure 5). Since *SWI4* is a G1/S target regulated by MBF, the resulting accumulation of Swi4 could drive this recruitment to further induce G1/S transcription. This would constitute a positive feedback loop involving Swi4. A similar positive feedback loop has been proposed to be important for G1/S transcriptional activation in mammalian cells [27]. The G1/S transcriptional activators E2F1, E2F2 and E2F3a, are G1/S targets and accumulate as a result of initial G1/S transcriptional activation. This coincides with further recruitment of these proteins to G1/S target promoters and activation of transcription. Additional research is required to establish the potential role of Swi4 in a positive feedback loop to activate G1/S transcription. If established, this would constitute a second positive feedback mechanism, in addition to the well established positive feedback of G1 cyclins [18], involved in ensuring robust activation of G1/S transcription that is likely conserved from yeast to humans.
